# Bleaching at acidic and basic pH for enamel and dentin whitening using internal and external techniques

**DOI:** 10.1590/1807-3107bor-2025.vol39.073

**Published:** 2025-07-07

**Authors:** Thais Regina Dias PAINI, Monique de SOUZA, Lidiane Vizioli de CASTRO-HOSHINO, Isolde Terezinha Santos PREVIDELLI, Antonio Carlos BENTO, Francielle SATO, Fernanda TSUZUKI, Raquel Sano Suga TERADA, Mauro Luciano BAESSO, Renata Corrêa PASCOTTO

**Affiliations:** (a) Universidade Estadual de Maringá – UEM, Department of Dentistry, Maringá, PR, Brazil.; (b) Universidade Estadual de Maringá – UEM, Department of Physics, Maringá, PR, Brazil.; (c) Universidade Estadual de Maringá – UEM, Department of Statistics, Maringá, PR, Brazil.

**Keywords:** Tooth Bleaching Agents, Dental Enamel, Hydrogen Peroxide

## Abstract

This study aims to evaluate in vitro the whitening effects of gels containing hydrogen peroxide at acidic and basic pH using internal and external techniques. Forty artificially darkened bovine incisors were split into four groups: bleaching on the buccal surface with HPE (HP Blue 35%, FGM), at basic pH; PBE (Potenza Bianco 38%, PHS), at acidic pH; bleaching in the pulp chamber with HPI at basic pH; and with PBI at acidic pH. CIELab color parameters, microhardness, roughness, enamel demineralization (FT-Raman), and surface topography (SEM) were evaluated in enamel/dentin blocks after bleaching (T0) and seven days after immersion in saliva (T7). Bleaching agent diffusion was evaluated after 30 minutes, while pH was measured throughout the application period. Physicochemical analysis showed a reduction in the mineral-matrix ratio in both groups after bleaching and at 7 days. Roughness increased over time in the PBE and HPE groups. Porosity increased, but decreased after 7 days of immersion in saliva. Bleaching gels were different at T0 and T7, with acidic pH gel promoting greater whitening (T7) and enamel roughness. Raman scanning demonstrated that the bleaches propagated across the enamel layer with increased concentration at the dentin-enamel-junction and decreasing diffusion gradient along the dentin. The gel with acidic pH promoted a greater increase in enamel roughness and the 7-day immersion in saliva was not enough to restore initial roughness in either gel. The gel with basic pH showed a higher pH decay and a greater diffusion capacity in the dentin.

## Introduction

Intracoronal bleaching has been considered a simple and less invasive procedure when compared to other treatments for non-vital teeth when there is color change and sufficient coronal structure to provide resistance to the remnant tooth.^1-3^

To shorten treatment time, in-office bleaching with hydrogen peroxide (H_2_O_2_) has been used at concentrations of 35% to 40%.^4^ However, high concentrations of hydrogen peroxide, as well as a more acidic pH, can cause structural changes such as increased surface roughness after bleaching, loss of mineral content, with consequent reduction in microhardness^5^ and higher risk of external cervical resorption.^6^

Research indicates that the concentration of H_2_O_2_ and the length of exposure to bleaching agents play a crucial role in the whitening effect; however, they are also associated with various adverse effects on dental tissues,^7,8^ including mineral loss,^8,9^ morphological alterations,^7,10^ and increased enamel roughness,^3,7^ with potential implications for the pulp tissue.^11,12^ Tooth sensitivity induced by bleaching typically emerges during the clinical procedure but tends to subside within 48 hours. It is widely recognized that this sensitivity stems from an inflammatory reaction triggered by H_2_O_2_ diffusion into the pulp, leading to the release of inflammatory mediators responsible for local vasodilation and increased vascular permeability.^13-15^ Therefore, while dental bleaching can enhance cosmetic outcomes, patients and practitioners should consider its associated risks to dental health and take measures to mitigate such risks.^13^

Although scientific research has shown a higher occurrence of enamel surface changes^16-17^ and prolonged postoperative sensitivity when using low-pH H_2_O_2,_^18^ it is essential to comparatively evaluate its whitening effect at acidic and basic pH, as well as of its impact on structural changes when applied to the enamel surface using the external technique.

Accordingly, this study aims to evaluate the whitening efficacy of gels containing H_2_O_2_at acidic and basic pH, applied through internal and external techniques, and their effects on enamel and dentin in vitro. The following null hypotheses were tested in this study: (a) the pH of the H_2_O_2_ gel would not influence its whitening efficacy; (b) the pH of H_2_O_2_would not influence the diffusion of bleaching agents through the tooth structure; (c) there would be no difference in the pH decay curve over time between bleaching agents with acidic and basic pH; and (d) there would be no difference between bleaching agents with acidic and basic pH in their potential to cause demineralization, increase surface roughness, or reduce enamel microhardness.

## Methods

### Experimental design

This study investigated the effects of different dental bleaching techniques and pH levels on various dependent variables. The fixed effects analyzed included the four experimental groups, determined by the combination of bleaching techniques (agents applied to internal or external surfaces) and pH levels (basic or acidic). On the buccal enamel surface, the treatments consisted of HPE, using HP Blue 35% (FGM) at a basic pH, and PBE, using Potenza Bianco 38% (PHS) at an acidic pH. In the pulp chamber, the treatments included HPI, bleaching at a basic pH, and PBI, bleaching at an acidic pH. The following dependent variables were evaluated: colorimetry, microhardness, roughness, enamel demineralization, surface topography, diffusion profile, and pH reduction. The teeth were randomly assigned to the experimental groups and subjected to standardized procedures to minimize bias and ensure the validity of the results. Evaluations were performed over time, allowing for a comprehensive analysis of the effects of the bleaching treatments.

**Figure 1:**
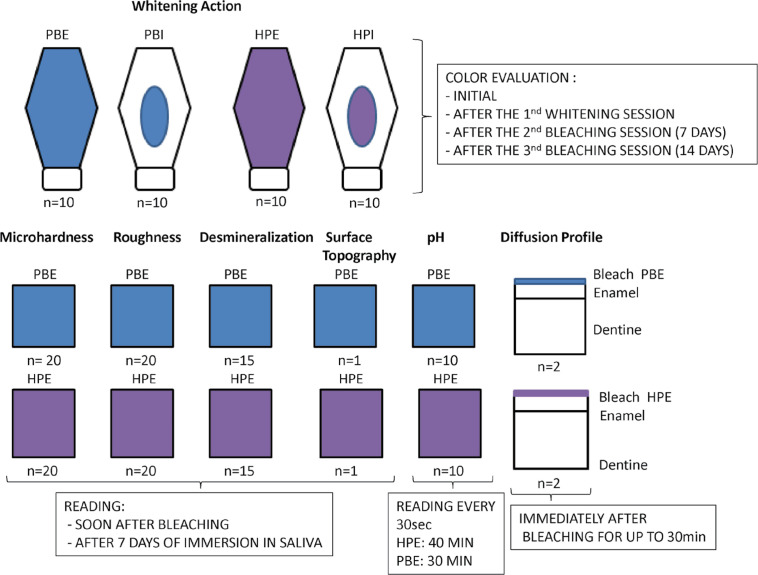
Study flowchart.

The volunteer who donated the blood for the tooth darkening signed an informed consent form to participate in the study, and ethical approval was obtained by the Ethics Committee in Human Research at the State University of Maringá, Paraná, Brazil (CAAE 14382313.9.0000.0104). Seventy bovine upper central incisors donated by a regulated and certified slaughterhouse were used. All teeth were obtained from the same batch of animals to ensure that they had similar ages and approximate degrees of dental calcification. The extracted teeth were sonicated, stored in distilled water under refrigeration, and randomly assigned to the techniques outlined in the study flowchart (Figure 1).

For the colorimetry evaluation (n = 40), the teeth were artificially darkened and randomly distributed into four groups (n = 10): HPI - bleaching gel with a basic pH applied to the pulp chamber; PBI - bleaching gel with an acidic pH applied to the pulp chamber; HPE - bleaching agent with a basic pH applied to the buccal surface; and PBE - bleaching agent with an acidic pH applied to the buccal surface. All groups were treated with three bleaching sessions, spaced 7 days apart. Microhardness (n = 20, HPE and PBE), roughness (n = 20, HPE and PBE), enamel demineralization (FT Raman, n = 15, HPE and PBE), and surface topography (SEM, n = 1, HPE and PBE) were evaluated on enamel/dentin blocks from bovine teeth before, immediately after bleaching (T0), and seven days after immersion in saliva (T7). The diffusion profile of the gels (cross-sectional Raman scanning, n = 4, HPE and PBE) was evaluated immediately after application of the bleaching agents after 30 minutes. The pH decay curve (n = 10, HPE and PBE) was measured every 30 seconds until the end of the application of each product. The study flowchart is shown in Figure 1. The bleaching gels with a basic pH (HP Blue 35%, FGM, Joinville, Brazil) and acidic pH (Potenza Bianco 38%, PHS, Joinville, Brazil) are detailed in Table 1.

**Table 1:** Bleaching agents, composition, pH, application method, and time.

Bleaching agents	Acronym	Composition and pH	Application method	Application time
Potenza Bianco PRO H_2_O_2_ SS	PB	38% hydrogen peroxide, thickeners, neutralizers, dyes, glycols, and deionized water. pH = 6	After connecting the tip of the syringe for phase A (38% hydrogen peroxide) with the syringe for phase B (thickener), the two phases were mixed by slowly pressing the plunger of one syringe back and forth 20 times (10x in each direction).	30 min
38%/PHS do Brazil (Joinville, SC, Brazil)				
Whiteness HP Blue/FGM (Joinvile, SC, Brazil)	HP	Active ingredients: 35% hydrogen peroxide (after mixing the phases). Inactive ingredients: Thickeners, blue pigment (HP Blue 20%), or violet pigment (HP Blue 35%), neutralizing agents, calcium gluconate, glycol, and deionized water. pH = 8.5	The two phases were mixed with the connected syringes, pushing the plungers alternately for up to 8 times (4x in each direction), pushing all the mixed contents into one of the syringes.	40 min

### Colorimetry

Teeth were darkened (n = 40) using the technique proposed by Paini et al.^19^to simulate staining caused by pulp necrosis. The pulp chamber received a hemolyzed blood solution derived from EDTA-treated whole human blood, which was centrifuged for plasma removal, followed by the addition of bidistilled water to promote hemolysis. Additional centrifugation for 20 minutes confirmed the rupture of all red blood cells. The pulp chamber was sealed with glass ionomer cement , and the teeth were immersed in artificial saliva for at 37ºC for 35 days, which was replaced every 24 hours.^19^ The darkened teeth were categorized into four experimental groups (n = 10 teeth/group) according to the bleaching agent and application technique (internal or external):

- HPE - basic pH bleaching agent applied to the vestibular surface (enamel);
- PBE – acidic pH bleaching agent applied to the vestibular surface (enamel);
- HPI - basic pH bleaching agent applied internally to the pulp chamber (dentin);
- PBI - acidic pH bleaching agent applied internally to the pulp chamber (dentin).

The roots of the artificially darkened teeth were sectioned 1 mm above the cementoenamel junction to facilitate the insertion of blood into the pulp chamber, using a 180° silicon carbide disc under refrigeration (Politriz, Arotec, Cotia, Brazil). The crowns were abundantly washed with air/water jets for 1 minute, followed by irrigation of the pulp chamber with 10 mL of 2.5% sodium hypochlorite and capped with temporary restorative material (Villevie, Joinville, Brazil) and resin composite (Charisma® Classic, Kulzer, São Paulo, Brazil), exposing the enamel surfaces. The teeth from the four groups were stored in saliva at 37°C in an oven throughout the experimental periods. Before each reading, the teeth were removed from the artificial saliva, gently dried with absorbent paper, and subjected to a whitening session, following the time intervals specified by the manufacturer. Subsequently, they were positioned in an individual silicon guide, which fit perfectly in the device and contained a hole in the middle third of the crown to standardize the reading area.

Color measurements were performed using the VITA Easyshade® Advance spectrophotometer (VITA Zahnfabrik H. Rauter GmbH& Co. KG, Germany), which generates the CIELab system — L* (brightness), a* (red-green axis), and b* (yellow-blue axis) — as well as on data from the CIELCH color system, as recommended by the Central Bureau of the International Commission on Illumination.^20^

Color variation ΔE was calculated using the Hunter formula.^21^ The resulting color changes in darkened teeth were analyzed based on the ΔE*ab distribution and classification system from the National Bureau of Standards (NBS): 1- 0.0 to 0.5 extremely slight change; 2- 0.5 to 1.5 slight change; 3- 1.5 to 3.0 noticeable change; 4- 3.0 to 6.0 marked change; 5- 6.0 to 12.0 extremely striking change; and 6- >12 severe color change.

The color difference, ΔE_00_, was calculated using L*, a*, and b* initial (darkened teeth) and final (bleached teeth) values, with the CIEDE2000 equation (Eq. 1)^22,23^


$$\Delta E_{00}=\sqrt{{\left(\frac{\Delta L^{\prime }}{K_{L}S_{L}}\right)}^{2}+{\left(\frac{\Delta C^{\prime }}{K_{C}S_{C}}\right)}^{2}+{\left(\frac{\Delta H^{\prime }}{K_{H}S_{H}}\right)}^{2}+R_{T}\left(\frac{\Delta C^{\prime }}{K_{C}S_{C}}\right)\left(\frac{\Delta H^{\prime }}{K_{H}S_{H}}\right)}$$ (1)

Where ΔL’, ΔC’ and ΔH’ represent the differences in lightness, chroma, and hue between the final and initial measurements, whereas R_T_ is a rotation function. S_L_, S_C,_ and S_H_ denote the weighting functions for the lightness, chroma, and hue components, respectively. K_L_, K_C,_ and K_H_ are the parametric weighting factors, all set to 1.^22,23^

WID (after bleaching) was calculated using the formula^24^ WID = 0.511L*-2.324a*-1.100b* and also, ΔWID = WID (after bleaching) - WID (before bleaching) to compare the index before and after the bleaching sessions.

### Fourier transform Raman spectroscopy (FT-Raman)

The teeth (n = 15) were split in half in the longitudinal direction using the gingival barrier (Top Dam, FGM, Joinville, Brazil), and in each half, a whitening gel was applied to the enamel. Readings were taken from the control tooth immediately after treatment and after seven days of treatment. The physicochemical alterations in enamel were evaluated using an FT-Raman instrument consisting of a Fourier-transform infrared spectrometer (Vertex 70v, Bruker Optik GmbH, Ettlingen, Germany) coupled to a Raman scattering detection module (Ram II module). The system operates with a Nd:YAG laser at a wavelength of 1064 nm and a germanium detector, maintained under liquid nitrogen cooling. A nominal power of 500 mW, 200 scans, a spectral resolution of 4 cm^-1^ (spectral range of 4,000 to 200 cm^-1^) were utilized. Data were baseline corrected and normalized by the total spectral area.

### Microhardness and roughness

For the microhardness and roughness tests, the specimens (n = 20 teeth/group) were cut into enamel blocks (4x4 mm) from the flattest portion of the buccal surface of the crowns on a water-cooled cutting machine (Isomet; Buehler, Lake Buff, United States of America). The specimens were then embedded in acrylic resin (Vipi Flash) and the entire buccal surface was polished using a polisher (Buehler, Lake Bluff, United States of America) under cooling. Silicon carbide sandpaper (Carbimet Buehler) was applied in descending grit sizes of #400, #600, and #1200, for 60 s. Between grit applications, specimens were cleaned with ultrasonic baths (Branson 2210) in deionized water for 2 min.

Enamel microhardness was determined using a Knoop diamond indenter (HMV 2000; Shimadzu, Tokyo, Japan) under a load of 50 g for 15 s. Three equidistant 100 µm indentations were made on the enamel surface in each specimen. Values were expressed in KHN. Surface roughness (Ra) was assessed with a profilometer (1700 Surf-Corder, Kosaka, Japan) equipped with a 5 μm radius diamond tip. In each reading, the needle traveled a 2.5 mm stretch on the surface at a constant speed of 0.5 mm/s, with a cut length of 0.25 mm. The average Ra (µm) of the specimens was obtained through three consecutive measurements, taken from the center of the specimen in different directions (45°).

### Hydrogen potential (pH)

Each tooth (n = 10 teeth/group) was inserted into a cylindrical tube, and 1.2 g of gel was applied to the buccal surface of the crowns. A pH meter electrode was placed in contact with the gel/tooth to monitor the pH variation. The pH of the bleaching gels as a function of application time was measured using a portable pH meter with digital display (mPA 210, Tecnopon, São Paulo, Brazil) with a precision of ± 0.005. Measurements were performed at room temperature (approximately 20°C), and electrode calibration was performed at pH 4, 7, and 10. The first pH reading was performed after 30 s of gel/tooth contact. Readings were taken every 30 s until the end of the application time indicated by the manufacturer. In the PBE group, 60 measurements were performed from 30 to 1,800 s (30 min). In the PEH group, 80 measurements were performed, from 30to 2,400s (40 min).

### Scanning electron microscopy (SEM)

Specimens (n = 1 tooth/group) were cut into enamel blocks (4x4 mm) from the flattest portion of the buccal surface of the crowns on a water-cooled cutting machine (Isomet; Buehler, Lake Buff, United States of America). Subsequently, the specimens were mounted on a metal holder with carbon tape and gold-sputtered to enhance surface conductivity. Microstructural analyses were performed under a scanning electron microscope (FEI Quanta 250, Thermo Fisher, United States) at 1000x magnification and a beam acceleration of 10 to 15 keV.

### Raman spectroscopy

Four blocks of bovine teeth (3 x 3 mm) were obtained with a natural enamel surface and a flat underlying dentin surface. The average specimen thickness was 2.8 mm. The specimens (n = 4 specimens/group) were individually fitted into a wax mold 7 (Asfer, São Caetano do Sul, Brazil) to guide the application of a 1 mm layer of bleaching gel (HPE and PBE) and also to ensure that it did not spread to the sides of the specimen surfaces. The whitening gel was applied on the external surface of the enamel and removed after 30 min with the aid of cotton swabs. The side of the specimen was then measured perpendicular to the bleached surface. The Raman spectra for evaluating the diffusion profile of the bleaching agents were obtained using a confocal Raman microscope (SENTERRA, Bruker Optik GmbH, Ettlingen, Germany). Readings were performed on the side of the specimen, starting from the enamel surface and extending into the dentin (cross-sectional Raman scanning), with measurement points placed every 100 µm up to approximately 2,800 µm. Excitation laser at 785 nm and nominal power of 100 mW were used. The laser was focused on the specimen using a 20x objective lens. Each spectrum represents an average of 30 scans with a 3s integration time and 3–5 cm^-1^ resolution in the spectral range of 1,778 to 419 cm^-1^. The spectra were baseline corrected and normalized by the total spectral area. To calculate the area, the spectra of the control specimens were subtracted from those of the bleached samples, thereby obtaining the signal solely from the bleaching band, centered at 873 cm^-1^.

### Statistical analysis

Results are presented as mean ± standard deviation (SD). For the FT-Raman analysis, the multivariate method was initially applied to the first derivative of all spectra, using principal component analysis (PCA), through a computer code developed in Mathematica 7.0 software (Wolfram Research, Champaign, United States), enabling the identification of the most prominent variables. Subsequently, descriptive statistics were applied to colorimetry, microhardness, roughness, FT-Raman, and pH data, , and given the nature of the longitudinal data, characterized by the dependency between observations taken over time, generalized linear mixed models (GLMMs) were used. More specifically, the normal model with a canonical link function, appropriate for the distribution of the response variable, was used. A random effect for the experimental unit (tooth) was included to account for the covariance structure between and within sample units, as recommended by Diggle et al.^25^ The significance level was set at 5%, and model adequacy was assessed through residual analysis, which confirmed the consistency of the adjustments. The paired Wilcoxon test was used to compare the pH decay curves of the bleaching gels, while SEM images were analyzed descriptively.

Descriptive statistics and graphs were performed using the summary function, and all models were fitted using R software version 3.2, utilizing the lme4 package.

## Results

### Colorimetry

Table 2 shows the behavior of the ΔE*ab color variation of the teeth immediately after the first bleaching session (T0), after 7 days of immersion in artificial saliva with the second bleaching session (T7), and after 14 days of immersion in artificial saliva with the third bleaching session (T14), using Potenza Bianco (PB) and Whiteness HP Blue (HP) applied with the internal (I) and external (E) techniques. The results revealed a significant difference (p = 0.01) in terms of color variation after three treatment sessions, with greater effect on color change at T7, when comparing the frequencies of occurrence of color change gradations (NBS5) between the HPI and PBI groups, as well as between the HPE and PBE groups and between HPI and PBE (p = 0.0001).

**Table 2:** Color assessment of darkened teeth (n = 10) using the CIELab system, as well as color differences obtained according to NBS parameters.

Group	L*	a*	b*	ΔE*ab	NBS 5	NBS 6
	(¯x ± SD)	(¯x ± SD)	(¯x ± SD)	(¯x ± SD)	Extremely striking	Color change
HPE	-36.9±11.4	4.3 ±2.7	-7.3± 3.7	38.1 ± 11.4	-	100
HPI	-46.4±7.1	7.8 ± 2.3	-7.2 ± 6.6	48 ± 7.3	-	100
PBE	-41.5± 16.2	7.3 ±5.2	-6.6 ± 5.6	43.1 ± 16.6	10	90
PBI	-45.5±11.4	4.6 ± 4.4	-6.7 ±7.3	46.9 ±11.2	-	100

**Figure 2:**
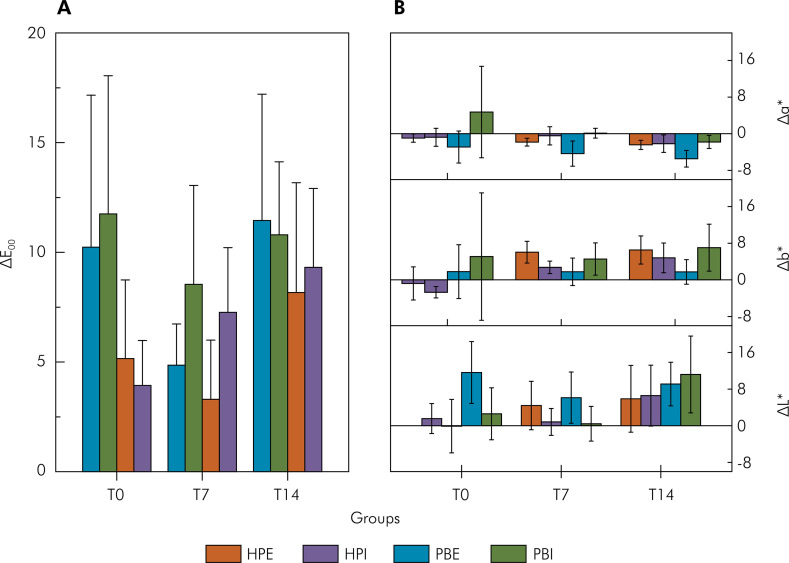
A) ΔE00 color variation of teeth immediately after the first bleaching session (T0), after 7 days of immersion in artificial saliva with the second bleaching session (T7), and after 14 days of immersion in artificial saliva with the third bleaching session (T14) using Potenza Bianco (PB) and Whiteness HP Blue (HP) gels applied with the internal (I) and external (E) techniques. B) variation of Δa*, Δb*, and ΔL* color coordinates after bleaching application: immediately (T0), 7 days (T7), and 14 (T14) days. Internal application of Potenza Bianco at acidic pH on the lingual (PBI) and buccal surfaces (PBE), and internal application of Whiteness HP Blue at basic pH on the lingual (HPI) and buccal surfaces (HPE). Each value represents the mean ± SD (n = 10 teeth/group).

Color variation, ΔE_00_*, was calculated using the CIEDE2000 color difference formula.^22^ Figure 2a shows the behavior of the ΔE_00_* color variation of the teeth immediately after the first bleaching session (T0), after 7 days of immersion in artificial saliva with the second bleaching session (T7), and after 14 days of immersion in artificial saliva with the third bleaching session (T14), using Potenza Bianco (PB) and Whiteness HP Blue (HP) applied with the internal (I) and external (E) techniques. The results indicated no significant difference in terms of color variation between the bleaching gels after three treatment sessions (p < 0.05). There was, however, a significant difference (p=0.01) at T7, with a higher effect on color change between the HPI and PBI groups, as well as between the HPE and PBE groups and between HPI and PBE (p = 0.0001).

Figure 2b shows the variation in color coordinates, Δa*, Δb* and ΔL*, comparing darkened specimens before the bleaching sessions and subsequent measurements at T0, T7, and T14 for internal and external applications of the bleaching gels. After bleaching sessions, the green color was predominant, Δa*< 0, except for HPI at T0. The yellow color prevailed after 7 days, Δb*> 0 for all treatments. Internal applications, HPI and PBI, demonstrated an increase in ΔL* after three bleaching sessions (T14).

### Fourier transform Raman spectroscopy (FT-Raman)

**Figure 3:**
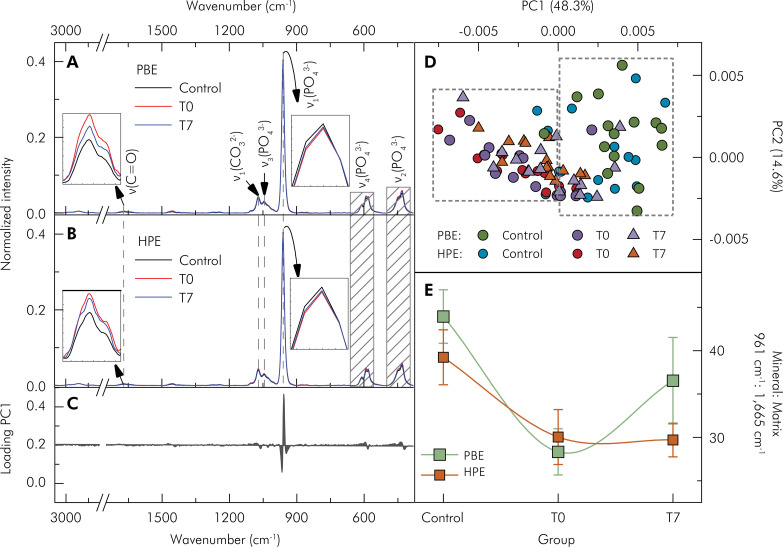
FT-Raman spectra of control teeth (Control) immediately after bleaching (T0) and after 7 days of bleaching (T7) with (A) Potenza Bianco (PBE) at acidic pH , and (B) Whiteness HP Blue (HPE) at basic pH . (C) Charge spectrum of the first principal component (PC1). (D) Principal component analysis (PCA) obtained from the first derivative of the Raman spectra. (E) Mineral matrix rate, obtained through the integral of the area under the curve of each band (961 cm-1 and 1,665 cm-1), each value representing the mean ± SD; (n = 15 teeth/group).

Figure 3 shows the FT-Raman spectra of control teeth (control) immediately after bleaching (T0) and after 7 days of bleaching (T7) with Potenza Bianco (Figure 3A) and Whiteness HP Blue (Figure 3B), highlighting the principal peaks^26^ The PCA method was applied to the first derivative of the spectra to distinguish the control from the post-bleaching groups. PC1 loading (Figure 3C) showed the greatest variation between the spectral data sets. The band centered at 961 cm^-1^, attributed to the phosphate group, significantly contributed to differentiating the spectra of the control groups from those of the post-bleaching groups (T0 and T7), as shown in the PC1 vs PC2 graph (Figure 3D). The mineral matrix rate was quantified by calculating the ratio of the area under the curve, obtained by integration of the band centered at 961 cm^-1^ and 1,665 cm^-1^, attributed to the phosphate group and C=O bonds related to amide I, respectively. The results showed a significant reduction in the mineral matrix rate of the groups, PBE and HPE, immediately after bleaching (p < 0.001) and after 7 days of bleaching (p = 0.001), compared to the control groups (before bleaching). There was no significant difference between the bleaching gels (p = 0.071). There was also no significant difference (p = 0.357) between T0 and T7. The mean and standard deviation of mineral matrix rate values of all experimental groups are shown in Table 3.

**Table 3:** Mean ± SD of mineral matrix content of enamel subjected to hydrogen peroxide with acidic pH (PBE) and basic pH (HPE) according to each evaluation time. Control group; T0: immediately after bleaching; T7: 7 days after bleaching.

Groups	Control	T0	T7
PBE	43.94 ± 3.081a	28.33 ± 2.665b	36.53 ± 4.993b
HPE	39.24 ± 3.168a	30.05 ± 3.169b	29.70 ± 1.936b

### Microhardness

**Figure 4:**
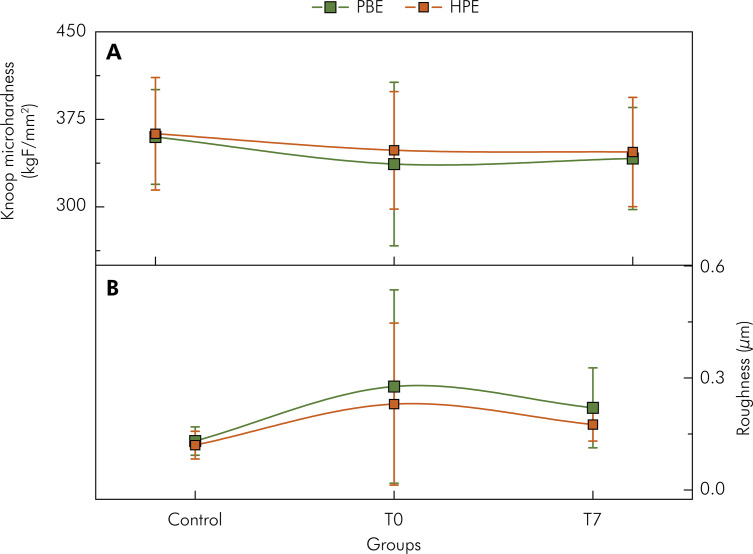
A. Knoop microhardness of control teeth immediately after bleaching (T0) and 7 days after bleaching (T7), with hydrogen peroxide at acidic pH (PBE) and basic pH (HPE). Each value represents the mean ± standard deviation; (n = 20 teeth/group). B. Roughness of control teeth immediately after bleaching (T0) and 7 days after bleaching (T7) with Potenza Bianco (PBE) at acidic pH and Whiteness HP Blue (HPE) at basic pH. Each value represents the mean ± SD; (n = 20 teeth/group).

Figure 4A shows the microhardness behavior of the teeth in their initial condition (control), immediately after bleaching (T0), and after 7 days of bleaching (T7) with Potenza Bianco (PBE) and Whiteness HP Blue (HPE). The means and standard deviations of the microhardness values of all experimental groups are shown in Table 4. The results of the inverse Gaussian model with log link indicated no significant difference between the main effects of treatment, HPE and PBE (p = 0.955), at the initial times, T0, and T7 (p = 0.322), and in their interactions (p = 0.550).

**Table 4:** Mean ± standard deviation of Knoop microhardness (KgF/mm2) of enamel subjected to hydrogen peroxide with acidic pH (PBE) and basic pH (HPE) according to each evaluation time. Control group; T0: immediately after bleaching; T7: 7 days after bleaching.

Groups	Control	T0	T7
PBE	359.90 ± 40.57a	336.69 ± 70.09a	341.45 ±4 3.7a
HPE	362.64 ± 48.17a	348.49 ± 50.34a	347.03 ± 4 6.80a

### Roughness

Figure 4B shows the roughness behavior of control teeth (control) immediately after bleaching (T0) and after 7 days of bleaching (T7) with Potenza Bianco (PBE) and Whiteness HP Blue (HPE). The means and standard deviations of the roughness values of all experimental groups are presented in Table 5. The results of the inverse Gaussian model with the logarithm link showed a significant increase in roughness in the PBE and HPE groups (p < 0.001) immediately after bleaching (p < 0.001) and 7 days after bleaching (p < 0.001) when compared to the control groups (before bleaching). There was a significant difference (p < 0.001) in the interactions of the bleaching gels at T0 and T7.

**Table 5:** Mean ± SD of roughness (μm) of enamel subjected to hydrogen peroxide with acidic pH (PBE) and basic pH (HPE) according to each evaluation time. Control group; T0: immediately after bleaching; T7: 7 days after bleaching.

Groups	Control	T0	T7
PBE	0.131 ± 0.038aA	0.277 ± 0.259bA	0.220 ± 0.107aB
HPE	0.120 ± 0.037aA	0.230 ± 0.217bB	0.175 ± 0.044bB

### Hydrogen potential (pH)

**Figure 5:**
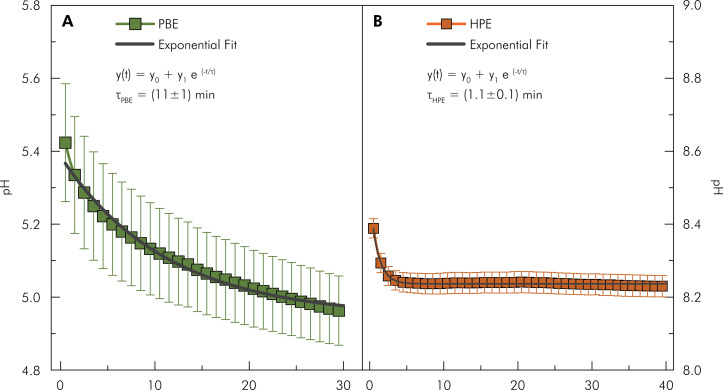
pH variation of Potenza Bianco (A) and Whiteness HP Blue (B) in contact with the tooth as a function of time. Each value represents the mean ± SD; (n = 10 teeth/group).

Figure 5 shows the pH behavior of the bleaching gel in contact with the tooth as a function of time. Note that PBE (Figure 5A) demonstrated gradual pH decline, following an exponential trend with a characteristic time of 10.98 min. HPE (Figure 5B) showed a fast pH decrease, exhibiting an exponential trend with a characteristic time of 1.10 min. The paired Wilcoxon test for the comparison between the initial and final time intervals showed a significant reduction (p = 0.005) in pH in the PBE group (n=10) as a function of contact time of the bleaching agent with the tooth, with a test power of 93% and a Cohen’s effect size of 0.887. A similar decrease occurred in the HPE group (p = 0.002). A faster pH reduction was observed in the HPE group (Figure 5).

### Scanning electron microscopy (SEM)

**Figure 6:**
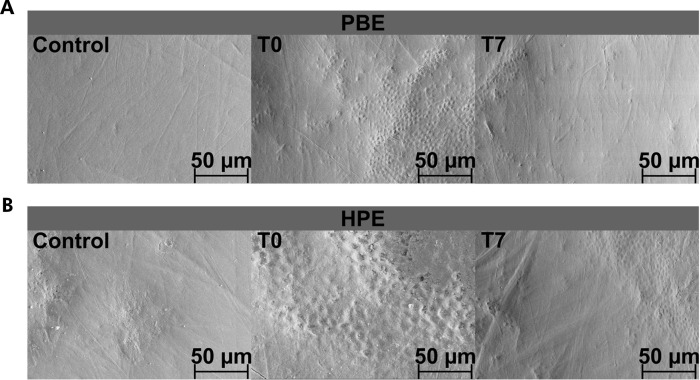
Scanning electron microscopy images (1,000x) for control tooth (Control) specimens, immediately after bleaching (T0) and after 7 days of bleaching (T7) with (A) Potenza Bianco at acidic pH, and (B) Whiteness HP Blue at basic pH ; (n = 1 tooth/group).

The surface of the specimens was analyzed by scanning electron microscopy (SEM) before bleaching, after bleaching, and after 7 days of immersion in saliva. No morphological changes were detected in the first group (without bleaching) during the experimental period. All micrographs show enamel surfaces at 1000x magnification (with the bar representing 50 µm). Signs of demineralization, with increased porosity, were observed on specimen surfaces shortly after bleaching. After 7 days, a reduction in porosity was noted in both groups. The micrographs are shown in Figure 6.

### Raman microscopy

**Figure 7:**
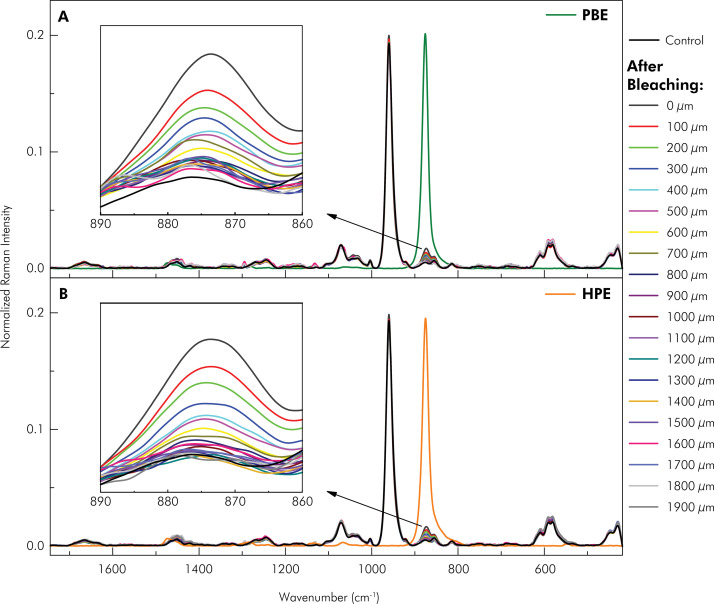
Raman spectra of control dentin and dentin after treatment with (A) Potenza Bianco (PBE) at acidic pH, and (B) Whiteness HP Blue (HPE) at basic pH, as a function of depth along the length of the specimen.

Figure 7 shows the Raman spectra of dentin as a function of depth after bleaching with Potenza Bianco (Figure 7A) and Whiteness HP Blue (Figure 7B). The intensity of the band centered at 873 cm^-1^, attributed to the O-O stretching,^27^decreases as the depth in the dentin increases. Depth zero µm corresponds to the region immediately after the dentoenamel junction. For better visualization, the data are presented up to a depth of 1,900 µm.

**Figure 8:**
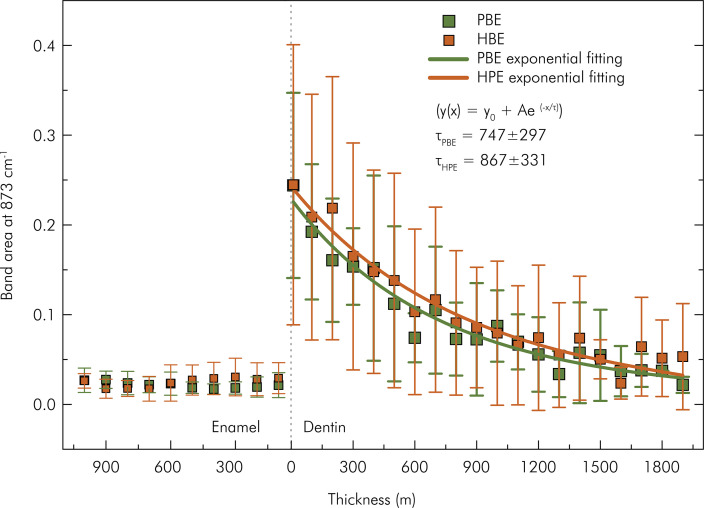
Behavior of the band area around 873 cm-1 as a function of depth in the specimen after application of Potenza Bianco (PBE) at acidic pH and Whiteness HP Blue (HPE) at basic pH; (n = 4 specimens/group).

The area under the curve at 873 cm^-1^was calculated to quantify the diffusion of the bleaching agents in the specimen. Figure 8 shows the diffusion profile of the bleaching agents. Note that the band area remained practically constant in the enamel layer. Beyond the dentoenamel junction, the area increased, exhibiting a decreasing behavior until reaching the final dentin thickness, suggesting retention of the bleaching agent in the dentin. An exponential decay function was applied to model the area variation for the dentin region. The adjustment estimated a characteristic thickness of 747±297 and 867±331 µm for Potenza Bianco and Whiteness HP Blue, respectively.

## Discussion

The first null hypothesis was partially rejected, given that the results showed a statistically significant difference in bleaching efficacy between the gels with acidic and basic pH applied through the internal or external techniques. A greater effect on color change was observed at T7 between the HPI and PBI groups, as well as between the HPE and PBE groups and between HPI and PBE (p = 0.0001). Both bleaching gels were effective after three sessions (T14). This finding confirms those found in randomized clinical trials.^18,28,29^

As observed in the WID formula, color coordinates a* and b* carry more mathematical weight than L*, considering our results exhibited more variation in a* and b* compared with L*, due to the artificially darkened teeth used. As a result, WID analysis may cause misinterpretation of the visual perception of whitening in teeth treated with bleaching agents. We believe that L*, in combination with a* and b*, can provide better assessment of the effects of bleaching gels on darkened teeth.

According to Ardu et al.,^30^ L* is sometimes considered the most important parameter to be analyzed because it measures the amount of black (L-) or white (L+) in a color, and it is directly linked to tooth brightness. However, the reported data show ΔE is not always accompanied by a significant increase in the values of L*, indicating that the color of the teeth is an interaction of different colors and that changes in a* and b* are also important for clinical evaluation and measurement of color after bleaching. This occurs because dental tissues, enamel, and dentin have different opacity and translucency characteristics that do not behave monochromatically, but instead involve the dispersion of different colors.^29^

For bleaching to occur, it is necessary for carbon chains of the chromogenic agents to be broken into simpler molecules, with less absorption and greater light reflection. Hydrogen peroxide is a highly unstable molecule and its decomposition reaction is influenced, among other factors, by the pH of the solution.^31^ Regarding bleaching gels, a large number of products have an acidic pH to increase their useful life, given hydrogen peroxide is more stable in an acidic medium. However, reducing the pH can promote enamel demineralization, changes in the chemical composition of the tooth structure, as well as a reduction in microhardness.^32^ On the other hand, clinical studies comparing bleaching agents with acidic and basic pH showed similar efficacy, but the basic gel caused less tooth sensitivity.^18^ This result may be due to the use of bleaching gels manufactured with different formulations, which may contain other ingredients in the formula, such as thickeners, fluorine, potassium, or nitrate (depending on the manufacturer), which may interfere more significantly with the final whitening outcome than pH alone.

In the present study, both bleaching gels provided significant whitening after three sessions (p < 0.05), with an interval of 7 days between sessions, revealing a lighter effect of the Potenza Bianco gel, with acidic pH at T7. Other studies have demonstrated that two in-office bleaching sessions led to a shift of approximately five shade units on the classic Vita scale.^18,33^ At first glance, the color change observed here seem inconsistent with those found by Torres et al.,^31^ who concluded that the efficacy of bleaching with hydrogen peroxide is directly proportional to the increase in the pH of the solution. Stoichiometric experiments have shown that perhydroxyl ion formation is influenced by pH. The higher the pH, the more ions are formed, leading to greater production of free radicals. The speed at which perhydroxyl ions are produced is closely related to the pH of the hydrogen peroxide solution.^31^ Likely, the faster decomposition of hydrogen peroxide in bleaching agents with basic pH can prevent or minimize the displacement of excess peroxide into the pulp chamber, where it could cause tooth sensitivity.

Additional evidence of this hypothesis can be found in the result of an in vitro study carried out by Mena-Serrano et al..^34^ The authors compared the penetration of hydrogen peroxide into the pulp chamber of different in-office bleaching gels and observed that such penetration was much more related to the application protocol and product employed than to the concentration of the bleaching agent. In that study, the presence of calcium significantly reduced the penetration of the bleaching agent into the pulp chamber.^34^ On the other hand, in the present study, the bleaching agent with basic pH (HP Blue), despite containing calcium in its formulation showed a greater diffusion capacity in the dentin, as evidenced by transverse Raman scanning . Notably, when hydrogen peroxide was incorporated into the thickener, formed a low-viscosity gel ,with bubble formation throughout application, demonstrating a high degree of oxidation. Conversely, the bleaching agent with an acidic pH (Potenza Bianco) formed a more viscous gel that remained stable on the surface throughout application time. This behavior may explain the results observed in the lateral Raman scan, which demonstrated the highest permeation of the bleaching agent at basic pH, despite the presence of calcium gluconate in the formulation. As a result, the second hypothesis was rejected. The greater permeation of the PB bleaching agent is further explained by the more pronounced reduction in the pH decay curve over time for the bleaching agent with basic pH, leading to the rejection of the third hypothesis.

In the present study, a silicon guide was used to position the spectrophotometer tip over the reading area during color measurements. This method would help to prevent color discrepancies resulting from variations in the placement of the spectrophotometer tip. However, recent studies have demonstrated that silicone guides can affect the accuracy of color readings and that their impact depends on the color of the guide.^35^ Santana et al. ^35^ found that repositioning guides had a minimal effect on L* values and no effect on b* values. The use of pink silicone increased a* values, whereas blue or translucent silicone reduced them. Notably, a reduction in data variability was specifically observed when colored silicones were used as guides, and it appears that tooth color measurement without any repositioning guide may be the most favorable approach. Moreover, the authors suggest that inaccurate color determination due to the presence of the repositioning guide may not necessarily impact the results observed in longitudinal studies. If the deviation from true colors remains consistent throughout a study, the difference between two different measurements is unlikely to differ significantly from a more accurate method of tooth color measurement such as a spectroradiometer.

In an in vitro study, Sun et al.^32^ also investigated the effects of 30% hydrogen peroxide with acidic (pH ≈ 3.6) and neutral (pH ≈ 7.0) pH on enamel, analyzing changes in color and in chemical structure, as well as mechanical properties and surface morphology. The present study revealed no significant difference in the efficacy of bleaching gels with neutral pH and acid pH after three sessions. The carbonate-to-mineral ratio and microhardness decreased more significantly with the use of the acidic gel than after the use of the bleaching agent with neutral pH, which, in turn, did not differ from the control group (distilled water). Significant morphological changes were also observed in the group bleached with the acidic pH gel.^32^ Therefore, the authors suggest that a bleaching agent with a neutral pH should be the first choice because 30% hydrogen peroxide with a neutral pH had the same efficacy in tooth whitening and promoted less deleterious effects on the enamel than its acidic pH counterpart.

The fourth null hypothesis was partially rejected, as both bleaching agents caused demineralization, increased surface porosity, and reduced enamel microhardness. On the other hand, the bleaching gel with acidic pH promoted a more significant reduction in enamel roughness compared to the bleaching gel with basic pH.

Manufacturers have expressed concern regarding the acidity of bleaching gels because a pH below the critical level (pH ≈ 5.5 to 6.5) can result in enamel dissolution, changes in microhardness, heightened sensitivity, increased enamel wear, and surface roughness.^5,17^ Bleaching gels that have an acidic pH tend to have a lower amount of calcium ions in their formulation. However, the inclusion of calcium in the formula allows reaching a neutral or basic pH, stabilizing the initial and final pH values and reducing the difference in these values, thus minimizing the deleterious effects associated with the bleaching treatment.^28^ This was not observed in the present study because no significant difference was noted between acidic and basic pH gels (containing calcium) in terms of surface demineralization and enamel microhardness.

While the present study evidenced greater roughness in the enamel treated with a bleaching agent with an acidic pH compared to a bleaching agent with a basic pH, immersion in saliva for 7 days was not able to restore surface roughness to initial levels for either group. However, microscopic evaluation showed that the application of both bleaching agents promoted an increase in enamel surface porosity, which was reduced after 7 days of immersion in saliva. This result is corroborated by other studies, which demonstrated that the presence of acquired pellicle and sufficient calcium, phosphate, and fluoride ions in the oral cavity may be responsible for maintaining the mineral content of enamel.^36-38^ The acquired enamel pellicle can neutralize the adverse effect caused by acid erosion^,39,40^given that calcium, phosphate, and fluorine ions have the potential to inhibit enamel demineralization and potentiate remineralization.^37,38,41^

Carrasco-Guerisoli^42^ demonstrated that the oxidation of H_2_O_2_ with the use of bleaching agents with acidic pH plays an important role in ultrastructural changes in the dentin during the internal bleaching of bovine teeth. On the other hand, the in-office use of basic products with reduced application time proved to reduce such morphological changes. According to the findings of the present study, no change was detected in the dentin after bleaching for 7 days, considering the signs of remineralization observed after contact with saliva.

In the present study, the lateral Raman analysis verified that the diffusion dynamics of the bleaching agents applied on the enamel followed the same pattern observed by Ubaldini et al..^27^ These authors correlated the ability of H_2_O_2_ to interact with dentin organic compounds with its penetration dynamics in dental tissues. Not only did the results provide new insights into the role of the chemical affinity of H_2_O_2_ during its diffusion through enamel and dentin, but they also demonstrated that H_2_O_2_ penetrated the enamel, reaching the underlying dentin layer, oxidizing the organic compounds and modifying its mineral composition.

The diameter and density of the dentinal tubules influence the permeability of the dentin.^43-45^ H_2_O_2_ applied to the enamel surface diffuses through the dentinal tubules.^46^ Thus, the accumulation of H_2_O_2_ in the outer layer of dentin could have resulted from the chemical affinity of H_2_O_2_ for the organic compounds of dentin, as well as from the reduced diameter of the dentinal tubules in this region.^27^

Other studies have suggested that the effect of H_2_O_2_on the relatively rich organic substance in the dentin can be attributed to action on collagen, leading to amino acid denaturation.^47-49^ This may explain the erosion pattern found in some studies, in which intertubular dentin, which is less mineralized, was more aggressively attacked than peritubular dentin.^42^

The present study sought to reproduce, under experimental conditions, the clinical procedures used for the bleaching of non-vital teeth. Nevertheless, one of the limitations was the use of artificially darkened teeth. Clinically, in cases of darkening due to pulpal necrosis, the presence of bacterial degradation products, as well as the length of time during which the tooth remains discolored in the mouth after pulpal death, could influence the success of the treatment.^50,51^

The difficulty in finding extracted human teeth, mainly anterior teeth, without caries, fractures, or cracks warranted the use of bovine teeth. Furthermore, bovine teeth are easier to bleach when compared to human teeth because of the larger diameter of dentinal tubules. Also, the use of bovine teeth has some advantages, especially for the ease of obtaining specimens that are at a similar stage of tooth development.^52^

Clinical studies testing the efficacy of these bleaching gels with acidic and basic pH on non-vital teeth may help to elucidate their effectiveness in treating darkening caused by pulp necrosis, aiding in the selection of a technique that provides faster and more comfortable bleaching outcomes.

## Conclusion

Our findings indicate no statistical difference at the 5% level between the bleaching gels with acidic and basic pH in terms of efficacy after three sessions and ability to promote superficial demineralization and enamel microhardness. Although the qualitative (microscopic) analysis showed that both bleaching gels increased enamel surface porosity, which decreased after seven days of immersion in saliva, the quantitative analysis demonstrated that the gel with acidic pH led to a larger increase in enamel roughness compared to the gel with basic pH. Moreover, seven days of immersion in saliva were not enough to restore the initial roughness values for either gel. The gel with basic pH showed a higher pH decay rate over time and a greater diffusion capacity in the dentin.

## Data Availability

After publication the data will be available on demand to the authors - condition justified in the manuscript.
